# Supplementary material for: Inhibition of BRD4 Promotes Pexophagy by Increasing ROS and ATM Activation
Source: Cells. 2022 Sep 12;11(18):2839. doi: 10.3390/cells11182839 (PMC9497081; doi:10.3390/cells11182839)
Supplement: Supplementary file 1 [file cells-11-02839-s001.zip › cells-1859745-supplementary.pdf]

cells

Inhibition of BRD4 Promotes Pexophagy by Increasing ROS and ATM Activation

Yong Hwan Kim [1,†], Doo Sin Jo [1,†], Na Yeon Park [1], Ji-Eun Bae [2], Joon Bum Kim [1], Ha Jung Lee [1], So Hyun Kim [1], Seong Hyun Kim [1], Sunwoo Lee [1], Mikyung Son [3], Kyuhee Park [4], Kwiwan Jeong [4], EunByul Yeom [1] and Dong-Hyung Cho [1,3,*]

[1] BK21 FOUR KNU Creative BioResearch Group, School of Life Sciences, Kyungpook National University, Daegu 41566, Korea
[2] Brain Science and Engineering Institute, Kyungpook National University, Daegu 41566, Korea
[3] Orgasis Corp., Suwon 16229, Gyeonggi-do, Korea
[4] Bio-Center, Gyeonggido Business & Science Accelerator, Suwon 16229, Gyeonggi-do, Korea
* Correspondence: dhcho@knu.ac.kr; Tel.: +82-53-950-5382.
† These authors contributed equally to this work.

Supplementary Figure

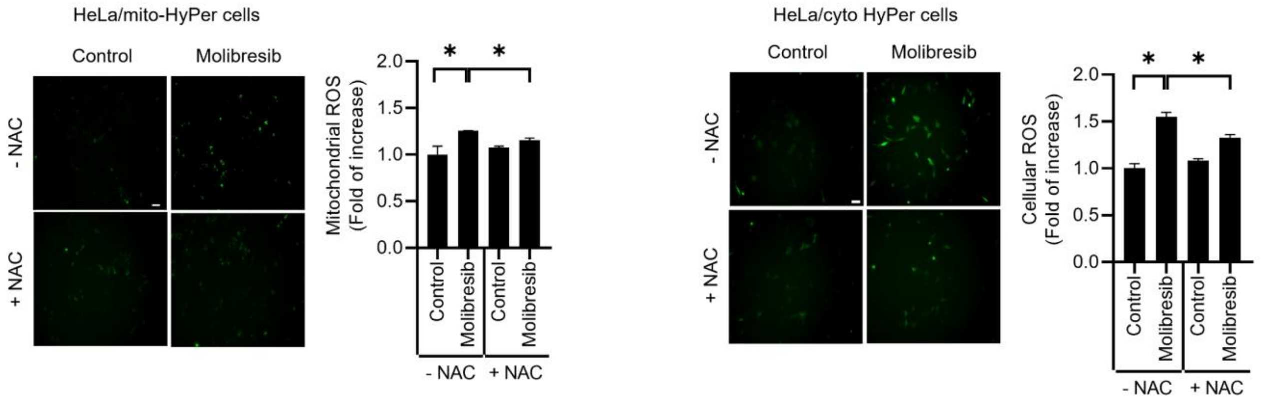

Supplementary Figure S1. Inhibition of BRD4 increases ROS levels in HeLa cells. HeLa cells expressing mitochondrial HyPer (HeLa/mito-HyPer) or cytosolic HyPer (HeLa/cyto-HyPer) were treated with molibresib (10 μM) with or without NAC (1 mM) for 48 h. The fluorescence intensity of HyPer protein were imaged and measured using image processing software ImageJ (the white scale bar, 50 μm). The experiments were repeated at least three times (Data indicate means ± S.E.M. *$p < 0.05$).
